# Surgical Cases

**Published:** 1889-12-14

**Authors:** 


### SURGICAL CASES.

In the *Birmingham Medical Review* for October, Mr. W. Haslam, F.R.C.S., assistant surgeon to the General Hospital, Birmingham, gives some interesting notes on surgical cases. Among them is mentioned "dermoid tumour of the nose." This occurred to a child aged three, on the tip of the nose, which is an extremely rare position for a tumour of the kind. An incision was made, and a quantity of white sebaceous matter squeezed out. It was impossible to dissect the sac away, so the interior was scraped and a small plug introduced. No hairs could be seen in the sac wall, nor could any be found on examining the fluid with the microscope. So-called dermoid tumours are often met with over the external angular process of the frontal bone, but also occasionally in other places, as over the nasal process of the superior maxillary bone, when they may be connected with the meninges of the brain. It may, however, be questioned if a tumour is rightly termed a dermoid tumour unless small hairs are found in the pultaceous contents.

The next case is congenital absence of the right fibula and the outer side of the foot. There was complete absence of the fibula on the right side, also of the two outer metatarsal bones, and their corresponding toes, while the second and third toes were webbed. The right tibia measured five inches, the left seven and a half, and there was most complete *talipes equinus*. The boy could run about very well, there being compensation for the want of length on the right side by some flexion of the left leg and dropping of the right side of the pelvis. The mother attributed the deformity to having been frightened by a horse during the early stage of pregnancy. In the "Retrospect," Hospital, October 26 last, we mentioned a case which had come to our personal knowledge of a child being born deformed, after a fright received by the mother.

